# Supplementary material for: Nanoscale Structure, Dynamics, and Aging Behavior of Metallic Glass Thin Films
Source: Sci Rep. 2016 Aug 8;6:30973. doi: 10.1038/srep30973 (PMC4976322; doi:10.1038/srep30973)
Supplement: Supplementary Information [file srep30973-s1.pdf]

Supplementary Information: Nanoscale Structure, Dynamics, and Aging Behavior of Metallic Glass Thin Films

J.A.J. Burgess,[†,‡,¶] C.M.B. Holt,[†,§] E.J. Luber,[†,‖] D.C. Fortin,[‡] G. Popowich,[‡] B. Zahiri,[†,§] P. Concepcion,[†] D. Mitlin,[†,§,⊥] and M.R. Freeman[*,†,‡]

†*National Institute for Nanotechnology, Edmonton, Canada*

‡*University of Alberta, Department of Physics, Edmonton, Canada*

¶*Now at the Max Planck Institute for Structure and Dynamics of Matter, Hamburg 22761, Germany*

§*University of Alberta, Department of Chemical and Materials Engineering, Edmonton, Canada*

‖*University of Alberta, Department of Chemistry, Edmonton, Canada*

⊥*Clarkson University, Chemical & Biomolecular Engineering, Potsdam, New York, United States*

E-mail: freemanm@ualberta.ca

Complementary Electron Microscopy

The dynamics observed on the CuHf films are only obvious with STM observations. However, in the interest of identifying the dynamics and potential aging as a fundamental process that is not completely attributed to tip interaction, electron microscopy observations were undertaken. High resolution SEM was used to confirm the structural changes of the films,

such as enlarging clusters during scanning or the larger spontaneous formation of very large surface clusters. Hopping clusters proved to be too small for clear observation with SEM. In order to verify hopping, the use of TEM is required, however the observations necessitate a change in substrate and film thickness.

Use of scanning electron microscopy offers the opportunity to observe films identical to those observed in the STM. A sample composed of $Cu_{80}Hf_{20}$, 50 nm thick was transfered into a Hitachi S-5500 high resolution SEM immediately following deposition onto an oxidized silicon wafer. The film structure reveals a very flat surface as expected, but also shows some sections where the topography exhibits larger smooth features protruding above the rest of the surface. The more variable topography in these sections makes the film structure less stable, meaning that these areas are ideal locations to investigate for dynamics.

Indeed, in these areas, multiple types of surface changes were seen. Upon scanning these features, the surface was found to planarize, and to convert from a smooth appearance into a pebbled texture. This corroborates the STM observations of aging. The SEM cannot image the extremely small amorphous clusters initially, however as scanning drives the unstable sections of the surface to rearrange, clusters grow and stabilize into the surface reconstruction observed with STM (Fig. 4). The clusters are lithographically enlarged with the beam, until they become large enough to be resolved by the SEM. This matches very well with the aging process observed following sputter cleaning of the STM samples.

Additional shifts of material in varying quantities between locations were observed (Fig. 5). Holes enlarged or filled in the film surface, large cluster like objects appeared and disappeared. Periodically, a preferential site would begin to accumulate material rapidly, forming very large surface features (Fig. 5). These events match the large surface changes found in STM, including the observation of material surrounding the large feature disappearing in conjunction with the feature growing.

The SEM observations did not have sufficient resolution to observe clusters sufficiently small that they exhibited stable hopping. Observations of films on TEM substrates (silicon

nitride, graphene, or carbon membranes) also proved to be challenging, with poor contrast when looking directly through the film. Success was found using an unconventional technique of depositing the sample films onto low density arrays of carbon nanotubes. Films were sputtered onto carbon nanotube substrates, coating the tubes with a very thin (5-15 nm) layer of $Cu_{80}Hf_{20}$. The samples were immediately transferred to a JOEL-2100 TEM for observation. The coating of a 3D substrate affords the opportunity to look at sections of the film in profile, where fluctuations in thickness of the film are much more obvious. A caveat to this observational technique is the fact that the film is on a different substrate, is a different thickness, and has very different film geometry. However, this approach did allow TEM based verification of hopping clusters as shown in figure 6 with hopping events highlighted by colored arrows.

STM Movies of Surface Dynamics

Movie 1: Successive STM scan frames of the $Cu_{81}Hf_{19}$ surface.

Movie 2: Successive STM scan frames of the $Cu_{50}Hf_{50}$ surface.

Movie 3: Successive STM scan frame series of the initially scanned $Cu_{75}Hf_{25}$ surface.

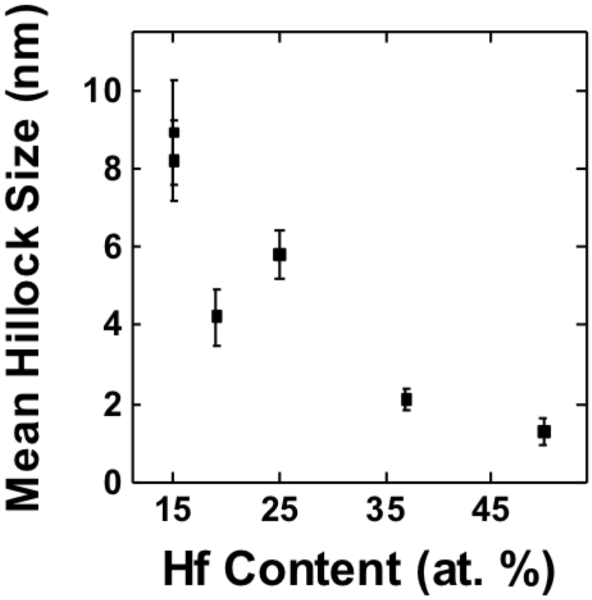

Figure 1: The mean hillock feature size decreases with increasing Hf concentration of $Cu_{1-x}Hfx$ films over the concentration ranges examined.

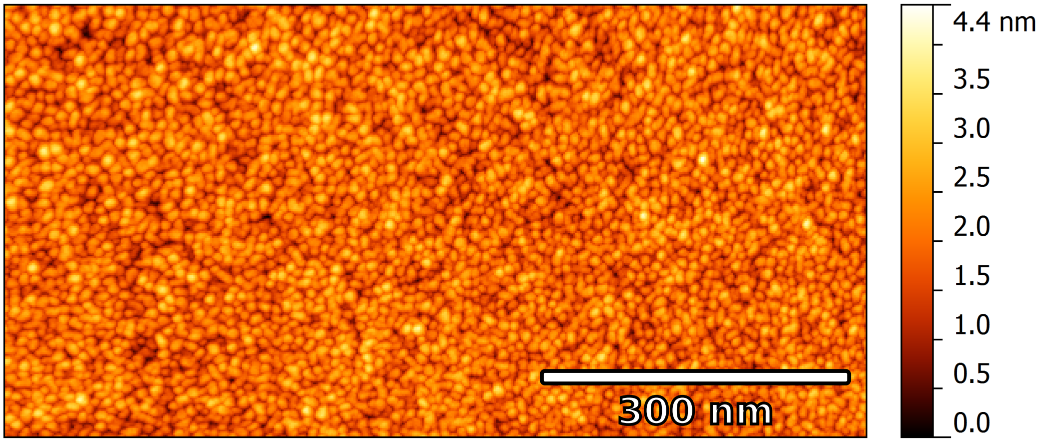

Figure 2: No hopping events are observable on $Cu_{85}Hf_{15}$ thin films with a thin coating of Au (10 nm). This image was acquired at 110 K. The scale bar is estimated using 4K and room temperature piezo calibrations in conjunction with the temperature response of PZT-5A.

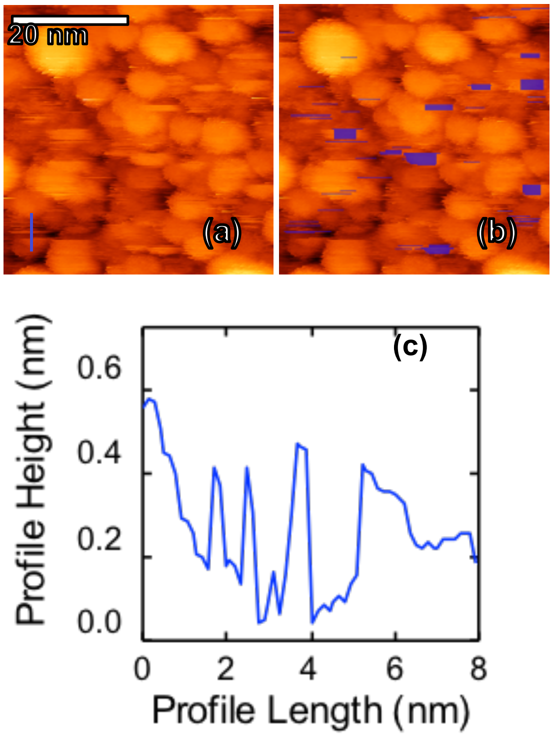

Figure 3: Hopping clusters lend a striped pattern to images created using raster scanning. An image of $Cu_{85}Hf_{15}$ shows numerous hopping clusters (a). Stripes corresponding to a cluster that is temporarily occupying a hopping site are marked in blue in panel (b). The width of these stripes, in scan lines, multiplied by the time per scan line yields the dwell time of the cluster. Acquiring a line profile (vertical blue line in panel a) over an active hopping site yields a telegraph noise-like trace showing the hopping site alternating between occupied and unoccupied.

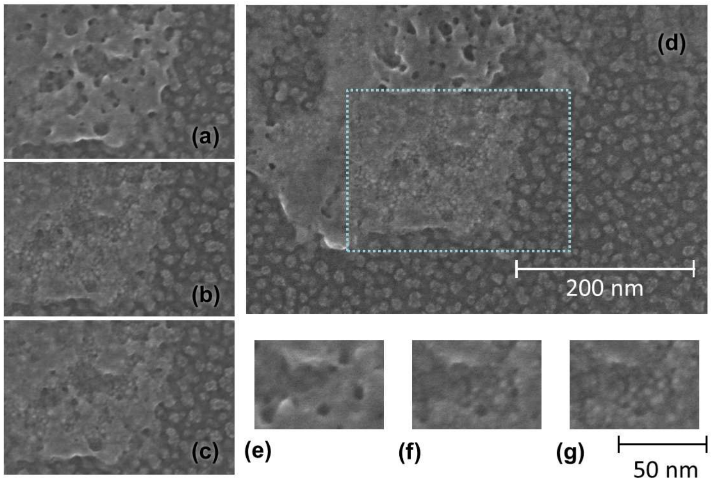

Figure 4: A reconstructed surface structure forms under the influence of the beam during SEM observations. Much as is seen in STM observations, within the scanning area pebble like surface features form, indicating the presence of clusters that enlarge steadily during observations (panels (a) through (c) are shown in order). In the case of SEM, this means the cluster features eventually become resolvable. Panel (d) offers a zoomed out view of the area where panels (a)-(c) were acquired, showing changes in the film are limited to the former scan area (at the center of the image). Note that panels (a)-(c) use the same scale as panel (d). Panels (e) through (g) provide a zoomed in view on the center region of panels (a) to (c). The conversion of the smooth surface features to a pebbled appearance is more clear with the increased zoom. The 50 nm scale bar applies to panels (e) through (g).

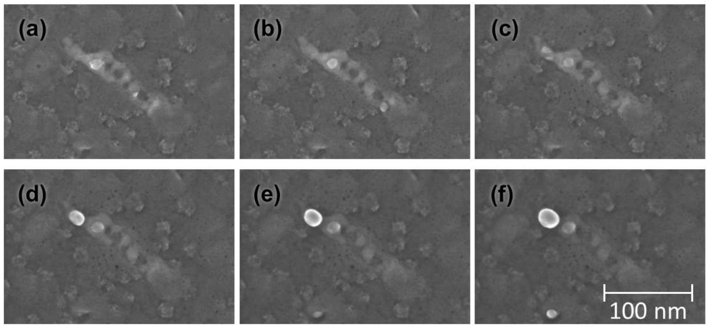

Figure 5: Rough sections of copper hafnium samples show dynamics observable with high resolution SEM. A large number of dynamical effects are visible over time (panels (a) through (f) are shown in order). Holes open up in formerly smooth sections of film. Large cluster like objects appear and disappear, and particular large clusters grow.

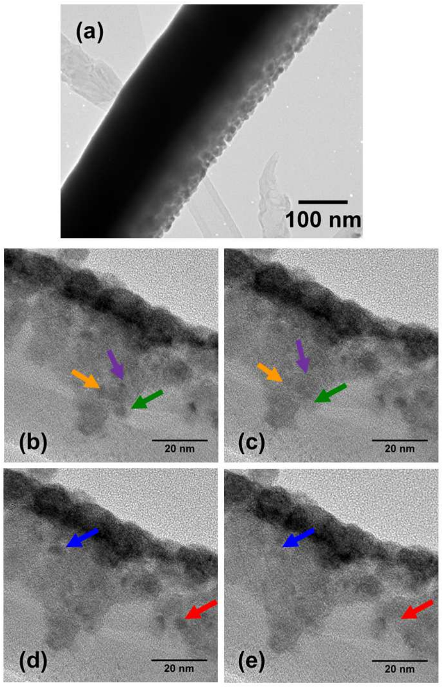

Figure 6: Under TEM observations of copper hafnium films in profile, occasionally nm scale sized clusters jump. Observations are made on films deposited on carbon nanotubes as shown in a zoomed out view in panel (a). At the edges of the tube, the film can be observed in profile. Jumps are rare, and difficult to spot. Fast TEM scans were acquired at a frequency of one per 60 s and compared to identify cluster jumps from image to image. In the four sequential panels shown, several clusters jump. Color coded arrows indicate transitions between frames.
